# ARP2, a Novel Pro-Apoptotic Protein Expressed in Epithelial Prostate Cancer LNCaP Cells and Epithelial Ovary CHO Transformed Cells

**DOI:** 10.1371/journal.pone.0086089

**Published:** 2014-01-22

**Authors:** Jaime Mas-Oliva, Enrique Navarro-Vidal, Juana Virginia Tapia-Vieyra

**Affiliations:** 1 Instituto de Fisiología Celular, Universidad Nacional Autónoma de México, México D.F., México; 2 División de Investigación, Facultad de Medicina, Universidad Nacional Autónoma de México, México D.F., México; II Università di Napoli, Italy

## Abstract

Neoplastic epithelial cells generate the most aggressive types of cancers such as those located in the lung, breast, colon, prostate and ovary. During advanced stages of prostate cancer, epithelial cells are associated to the appearance of androgen-independent tumors, an apoptotic-resistant phenotype that ultimately overgrows and promotes metastatic events. We have previously identified and electrophysiologically characterized a novel Ca^2+^-permeable channel activated during apoptosis in the androgen-independent prostate epithelial cancer cell line, LNCaP. In addition, we reported for the first time the cloning and characterization of this channel-like molecule named apoptosis regulated protein 2 (ARP2) associated to a lethal influx of Ca^2+^ in *Xenopus* oocytes. In the present study, LNCaP cells and Chinese hamster ovary cells (CHO cell line) transfected with *arp2-*cDNA are induced to undergo apoptosis showing an important impact on cell viability and activation of caspases 3 and 7 when compared to serum deprived grown cells and ionomycin treated cells. The subcellular localization of ARP2 in CHO cells undergoing apoptosis was studied using confocal microscopy. While apoptosis progresses, ARP2 initially localized in the peri-nuclear region of cells migrates with time towards the plasma membrane region. Based on the present results and those of our previous studies, the fact that ARP2 constitutes a novel cation channel is supported. Therefore, ARP2 becomes a valuable target to modulate the influx and concentration of calcium in the cytoplasm of epithelial cancer cells showing an apoptotic-resistant phenotype during the onset of an apoptotic event.

## Introduction

Carcinomas that find their origin in epithelial cells are the most common type of cancer in adults and make up to 80–90% of all cancers, including those located in the lung, breast, prostate, ovary and colon. Epithelial cells among other localizations, line internal cavities and form the lining of glandular tissues. The prostate gland is mainly composed of epithelial cells and interstitial stromal cells that communicate between themselves and control, not only the normal development of the gland, but also the onset of neoplastic growth [1]. Prostate cancer is one of the most frequently diagnosed noncutaneous cancers. The initial stages of prostate cancer progression depend on androgens that increase proliferation and inhibit apoptosis. Therefore, androgen-deprivation therapy has been the major course of treatment in recurrent and metastatic prostate cancer. Prostate epithelial cells that are primarily luminal include a mixture of several cell types including basal and neuroendocrine cells [2], [3], while adjacent stromal cells, composed of a mixture of fibroblasts, nerve, and smooth muscle cells [2], [4], [5], intervene in the development of epithelial cancer cells and affect their response to hormones [6]. During the process of carcinogenesis, although the number of neuroendocrine cells increases in the more advanced stages of prostate cancer, epithelial cells representing the main tumor cell type are in the greater extent responsible for androgen-insensitive cell proliferation [7]. These cells present an apoptosis-resistant phenotype, do not undergo apoptosis in response to physiological stimuli [8], [9], and therefore are unresponsive to conventional chemotherapeutic agents [10]–[13].

Meanwhile, epithelial ovarian cancer has been found to be the sixth most common cancer in women with the highest rates being observed in northern european countries and the USA, whereas the lowest rates are found in the developing world. Due to the fact that pathogenesis of epithelial ovarian cancer is poorly understood, early detection has been mainly unsuccessful and new therapeutic approaches scarce. Since epithelial ovarian cancer cells have also been found to show an apoptosis-resistant phenotype due to the overexpression of anti-apoptotic genes such as Bcl-2, Bcl-xl and Survivin [14], the discovery and activation of new pro-apoptotic pathways has been an important approach to control proliferation and growth of this transformed cell type [15].

Apoptosis, a type of programmed cell death [16]–[20], has been shown to be of critical importance in cell homeostasis, embryonic development and several diseases such as cancer [21], [22]. Apoptosis is activated by cell-type specific mechanisms [16], [22], and occurs in several stages [19], [21], [23], [24]. The first stage is related with internal or external stimuli, while the second includes signal transduction mechanisms. The third stage is constituted by execution mechanisms that involve the proteolytic activity of caspases, a biochemical base for the apoptotic phenotype [25]–[28], and finally, the fourth stage that corresponds to condensation of nuclear chromatin, nuclear fragmentation and phagocytosis of apoptotic bodies by neighboring cells or macrophages [24], [29]. Overall, a sustained increase in [Ca^2+^]i has been shown to activate a series of cytotoxic mechanisms associated with apoptosis in different cell types [30], [31]. Since apoptosis has been proposed to be a mechanism that regulates tumor growth, the modulation of apoptosis activating mechanisms has therefore been considered as a valuable target to control the proliferation and growth of epithelial cancer cells [32]–[36]. In this respect, depending on the condition of the plasma membrane of transformed cells and the amount of extracellular calcium that enters the cytoplasm, a fine balance is achieved between the onset of an apoptotic event and/or the activation of a necrotic death pathway [37], [38].

In response to two different inducers of apoptosis; ionomycin and serum deprivation, we have previously shown based on electrophysiological findings, the activation of a Ca^2+^ permeable, non-selective cation channel in prostate epithelial LNCaP cells [15]. To further investigate these results, a series of Ca^2+^-permeable channels expressed during apoptosis were analyzed [15], [39]–[41]. During the course of these studies, two cDNAs, *arp1* and *arp2*, corresponding to two molecules synthesized during apoptosis induced by serum deprivation, were cloned. When *arp2* mRNA was injected into *Xenopus laevis* oocytes, ARP2 expression was induced followed by an influx of Ca^2+^ across the cell membrane and induction of apoptosis [40].

Taking into account that most epithelial cells share tissue organization characteristics as well as mechanisms involved in tumorogenesis [42], the present study shows that *arp2* cDNA transfection carried out using the original epithelial prostate cancer cells (LNCaP cell line) from which the pro-apoptotic calcium channel was described, promotes cell death through an apoptotic process when cell viability and caspases activation are measured. In order to make evident that the apoptotic initiation and attainment mechanisms are shared by diverse epithelial cells, our study has been extended to the transfection with *arp2* cDNA of epithelial ovary transformed cells (CHO cell line). Results shown in the present study support our previous findings and hold up the notion that ARP2, a novel calcium channel placed in the plasma membrane of cells during an event that might compromise cell viability and would lead to apoptosis, could be considered as a valuable new target to control the growth of the most aggressive epithelial cancer cell types.

## Materials and Methods

### Materials

The human androgen-insensitive prostate cancer cell line, LNCaP, and the Chinese hamster ovary cell line, CHO (*Cricetulus griseus*), were obtained from American Type Culture Collection (ATCC, Manassas, VA, USA), where they are regularly verified by genotypic and phenotypic tests. Original LNCaP cell lines received from ATCC included androgen-sensitive and androgen-insensitive cells. During the course of their use along the years, they have presented differentiated responses to growth depending on the presence or the absence of androgen analogs. All reagents for cell culture media were obtained from Gibco BRL (Gaithersburg, MD, USA). Vectors that were used included: pcDNA 3.1/V5-His-TOPO vector (Invitrogen, Carlsbad, CA, USA), pcDNA3.1/V5-His-TOPO *lacZ* vector (Invitrogen, Carlsbad, CA, USA), and the pEGFP-N1 vector (Clontech, Mountain View, CA, USA). Lipofectamine and Fura-2/AM were received from Invitrogen/Life Technologies Corporation (Carlsbad, CA, USA). Bis-acrylamide, Nonidet-P40, ethidium bromide, aprotinin, phenylmethylsulfonyl fluoride (PMSF), benzamidine, dimethyl sulphoxide (DMSO), ionomycin and dithiothreitol (DTT) were obtained from Sigma (St. Louis, MO, USA). r*Tth* DNA polymerase XL was obtained from Roche Molecular Systems (Branchburg, NJ, USA) and deoxyribonucleotide dNTPs were obtained from Boehringer Mannheim-GmbH (Mannheim, Germany).

### Plasmid construction for ARP2 expression

For amplification of *arp2* cDNA, 20 picomolar of a sense primer (5′-TGACAGTGATGCGGGAGAAGG-3′) and an antisense degenerate primer that corresponds to a conserved region of the Trp family of proteins (5′-TGY-TCK-MGC-AAA-YTT-CCA-YTC-3′) were used [40], [43]–[45]. PCR reactions also included 10 ng/mL linearized pCR 4Blunt-TOPO-ARP2 plasmid, 10× buffer, 25 mM MgCl_2_, and 10 mM dNTPs. The following cycles were performed: 94°C for 5 min, 30 cycles of 94°C for 45 s, 65°C for 45 s, 72°C for 2 min, and a final cycle at 72°C for 10 min. PCR products were separated and visualized in 1% w/v agarose gels containing ethidium bromide, and bands were excised and purified using a Concert gel extraction system (Gibco, Gaithersburg Maryland). PCR products were cloned into the pcDNA 3.1/V5-His-TOPO vector then propagated in *E. coli* DH5α competent cells (American Type Culture Collection, Manassas VA, USA). The plasmid obtained was named pcDNA3.1 ARP2 V5-His. The cDNA that codifies for enhanced green fluorescent protein (*eGFP*) was then inserted between the *Xhol* and *Xbal* sites of the pcDNA3.1 ARP2 V5-His plasmid, thereby generating the pcDNA3.1 ARP2-eGFP V5-His plasmid.

### Cell culture and transfections for transient expression

Androgen-insensitive LNCaP cells and CHO cells were cultured in RPMI 1640 and DMEM/F-12 medium, respectively. These culture media were also supplemented with 10% v/v fetal bovine serum (FBS), 2 mM L-glutamine, 0.2% w/v sodium bicarbonate, and 1% v/v penicillin-streptomycin. Cultures were maintained at 37°C and 5% CO_2_ until cells reached 60–70% confluence. Apoptosis was induced by incubating cells with culture medium deprived of FBS for different periods of time. Ionomycin was prepared as 5 mM stock solutions in DMSO. Ionomycin at a final concentration of 10 µM was applied to CHO and LNCaP cells cultures and incubated for different periods of time. Both cell lines were transfected with pcDNA3.1 ARP2 V5-His (Transfection efficiencies: TE/LNCaP 32%; TE/CHO 46%) and pcDNA3.1/V5-His-TOPO *lacZ* plasmids (TE/LNCaP 43%; TE/CHO 54%) to induce transient expression of ARP2-V5 and β galactosidase-V5, respectively. For normalization of cell viability assays, both cell lines were also transfected with plasmid pcDNA3.1 V5-His (without *arp2* cDNA) (TE/LNCaP 68%; TE/CHO 80%). CHO cells were transfected with pEGFP-N1 (TE 82%) and pcDNA3.1 ARP2-eGFP V5-His plasmids (TE 50%) to obtain eGFP and ARP2-eGFP expression, respectively. Transfections were performed using Lipofectamine 2000 as described in the pcDNA3.1/V5-His TOPO TA Expression Kit insert from Invitrogen (Carlsbad, CA, USA).

### [Ca^2+^]i measurements in whole cell suspensions using Fura-2

Androgen-insensitive LNCaP cells and CHO cells cultured as previously described, were removed from culture dishes using harvest buffer containing 10 mM HEPES-buffered 0.9% saline plus 0.05 EDTA, pH 7.4 according to Hirst et al. [46]. Cells are placed in suspension, and based on the same method sedimented at 500× *g* in a low-speed centrifuge for 3–5 min and rinsed twice with Krebs HEPES buffer containing 140 mM Na^+^, 4.7 mM K^+^, 1.3 mM Mg^2+^, 125 mM Cl^−^, 25 mM HCO ^3–^, 1.2 mM H_2_PO^4–^, 1.2 mM SO_4_^2–^, 10 mM glucose, 0.1 mM EGTA and 10 mM HEPES, pH 7.4. Supernatants were removed and pellets resuspended with Krebs HEPES buffer. A Fura-2/AM (3 µM) loading time of 30 min was carried out using Krebs HEPES buffer at 37°C in the dark. After this procedure is completed, cells were sedimented at 500× *g*, resuspended in Krebs HEPES buffer and further incubated for 20 min at room temperature to allow for de-esterification of Fura-2/AM.

After treatment, cells were twice sedimented at 500× *g* for 3 min and resuspended in Krebs HEPES-Ca^2+^ buffer (omitting EGTA and added of 2.5 mM Ca^2+^). Fura-2/AM was prepared as a stock solution (1 mM) by dissolving in dimethylsulfoxide and aliquots (10 µL) stored at −20°C until required.

Cell suspensions were maintained on ice and for each experiment placed in quartz cuvettes and incubated for 2 min at 37°C before measurements took place. A SLM-Aminco spectrofluorometer (Rochester, NY) was employed using an excitation ratio of 340/380 nm and maximum fura-2 fluorescence emission values at 510 nm. Calibration of fluorescence was carried out by the addition of 0.1% Triton X-100 to produce cell lysis liberating fura-2 into the Ca^2+^ or EGTA containing media. Maximum and minimum fluorescence values were substituted into the Grynkiewicz, Poenie & Tsien equation to calculate [Ca^2+^]i [47].

### Western blot analysis

Transient expression of ARP2 and β-Gal was achieved in LNCaP cells and CHO cells by transfection with *arp2* cDNA and *lacZ* cDNA, respectively. These proteins were expressed as fusion proteins with the V5 epitope at the carboxyl terminus of each (Invitrogen, Carlsbad, CA, USA). Cells were cultured for 16, 24, 48, and 72 h, then harvested and lysed with the following buffer: 10 mM Tris-HCl (pH 8.0), 100 mM NaCl, 1 mM EDTA, 1 µg/mL aprotinin, 1 mM PMSF, and 5 mM benzamidine. Protein quantification was performed using a bicinchoninic acid method (BCA) (Pierce, Rockford, IL, USA), and 20 µg of total protein were electrophoresed in 10% SDS-PAGE gels. Proteins were subsequently electroblotted onto 0.45 µm nitrocellulose membranes (Trans-Blot Transfer medium BIO-RAD, Hercules, CA, USA), then blocked for 1 h at 37°C with a “saturating” solution [Tris-buffered saline (TBS) (pH 7.6), 20 mM Tris-HCl (pH 7.4), 100 mM NaCl, 0.1% v/v Tween-20, and 2.5% w/v skimmed milk]. Primary anti-V5 monoclonal antibody (Invitrogen, Carlsbad, CA, USA) was diluted in the same saturating solution (1∶5000) and incubated with membranes for 12 h at 4°C. After membranes were washed three times with saturating solution at 37°C (15 min each wash), membranes were incubated at 37°C for 2 h with anti-mouse secondary antibody (Santa Cruz Biotechnology, CA, USA) diluted in saturating solution (1∶5000). Membrane were then washed with TBS/0.1% v/v Tween-20 three times for 15 min at 37°C, followed by a fourth wash with TBS at 37°C. Equal loading was verified by incubating the membranes with anti β-actin antibody (Santa Cruz Biotechnology, CA, USA). Visualization of antibody binding was achieved using an enhanced chemiluminescence (ECL) detection kit (Pierce, Rockford, IL, USA) and exposure of membranes to radiographic films (Kodak, Rochester, NY, USA).

### 3-(4,5-dimethylthiazol-2-yl)-2,5-diphenyl tetrazolium bromide (MTT) assay

Untreated cells were maintained in culture medium supplemented with serum (negative control), while apoptotic cells were obtained by serum deprivation (positive control). Cells (2×10^4^/well) were plated in 96-well plates and maintained in culture for 16, 24, 48, and 72 h. Then, 30 µL of MTT stock solution (Sigma-Aldrich, St Louis, USA) was added to each well to a final concentration of 0.5 mg/mL reagent. Plates were incubated at 37°C for 4 h to allow the growth of formazan crystals, which were subsequently solubilized with the addition of lysis buffer [20% SDS, 50% N, N-dimethylformamide (pH 3.7)]. Plates were incubated for 12 h, and absorbance values were measured at 570 nm using a microplate reader. Viability in cells transfected with plasmid pcDNA3.1-V5-His was considered for normalization of viability results of pcDNA3.1 ARP2 V5-His transfected cells. Each experiment was repeated three times (n). The Student's two-tailed *t-*test was used to determine statistical significance of differences with respect to controls. Data are expressed as mean X ± SEM and *p<*0.05 was considered as significant. Symbols denote: ^#^ *p*<0.05, *p<0.01, ^¶^ *p*<0.001

### Trypan blue cell viability assay

LNCaP cells were incubated for 16, 24, 48, 72, 96, and 120 h, while CHO cells were incubated for 16, 24, 48, and 72 h. Cells were stained with 0.1% v/v trypan blue dye then placed in Neubauer chambers. A total of 300 cells were counted for each sample using a Motic bright field optical microscope. Viability of cells transfected with plasmid pcDNA3.1-V5-His was considered for normalization of pcDNA3.1 ARP2 V5-His transfected cells. Each experiment was repeated at least three times (n). The Student's two-tailed *t-*test was used to determine statistical significance of differences with respect to controls. Data are expressed as mean X ± SEM and *p<*0.05 was considered as significant. Symbols denote: ^#^ *p*<0.05, *p<0.01, ^¶^ *p*<0.001

### Activity measurements of caspases 3 and 7

CHO and LNCaP cells (3×10^5^/well) were plated and cultured for 16, 24, 48, and 72 h. Cells were washed with PBS, harvested, and subjected to four freeze-thaw cycles in a lysis buffer (10 mM HEPES (pH 7.0), 40 mM b-glycerophosphate, 50 mM NaCl, 2 mM MgCl_2_). Samples were centrifuged for 30 min at 15 800 *x* g. Protein concentration for the supernatants collected were determined using the BCA method (Pierce, Rockford, IL). Samples (total protein, 20 µg) were diluted in assay buffer (50 mM HEPES, 10% sucrose, 0.1% CHAPS, 10 mM DTT) to a final volume of 1000 µL. The assay buffer was also supplemented with Z-Asp-Glu-Val-Asp-7-amino-4-trifluoromethylcoumarin (Z-DEVD-AFC), a fluorogenic substrate of caspases 3 and 7, and samples were incubated for 10 min at 30°C. Fluorescence was measured according to a Molecular Probes protocol using a luminescence spectrometer. The excitation wavelength was 365 nm and the emission wavelength was 505 nm. The maximum absorbance wavelength was found to be 488 nm. Fluorescence was measured in cell-free extract blank samples in order to detect the signal-to-noise ratio of the samples. Each experiment was repeated at least three times (n). The Student's two-tailed *t-*test was used to determine statistical significance of differences with respect to controls. Data are expressed as mean X ± SEM and *p*<0.05 was considered as significant. Symbols denote: ^#^ *p*<0.05, *p<0.01, ^¶^ *p*<0.001

### Confocal microscopy and differential interference contrast microscopy (DIC)

Confocal images were obtained using a Spectral Fluoview FV 1000 laser scanning confocal system (Olympus Corporation, Tokyo, Japan) attached/interfaced with an Olympus IX81 inverted light microscope with 10× and 40× oil-immersion objectives. eGFP was excited at 488 nm using a krypton/argon laser line and emitted fluorescence detected between 515 nm and 540 nm using a bandpass filter. Laser operation was performed at low power (97–99% attenuation) which substantially reduced photobleaching and photodamage to specimens. DIC microscopy images were obtained using an Olympus IX81 microscope and a IX2-LWUCD capacitor with 10× and 40× objectives. Confocal and DIC images were visualized, processed, and converted to TIFF formatted images using the FV10-ASW 6.1 software (Olympus).

## Results

### ARP2 expression and cell viability in LNCaP cells

**Figure 1:**
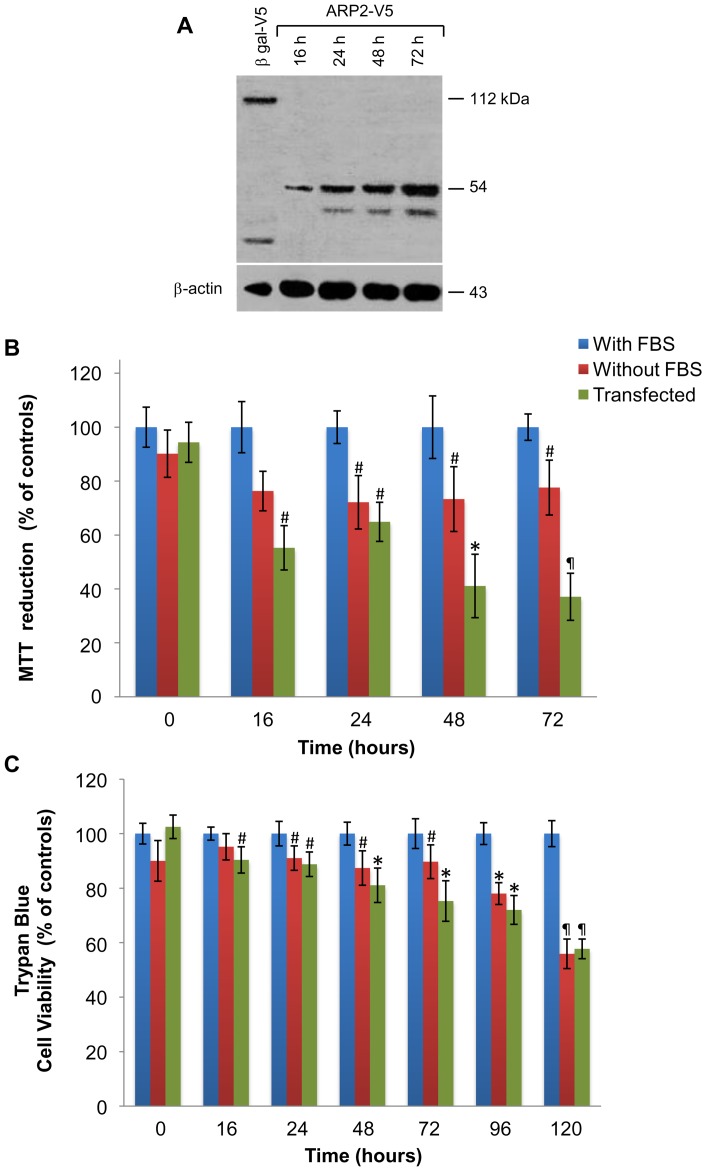
ARP2 expression induces apoptosis in LNCaP cells. (**A**) Western blots detected expression of β-gal-V5 (112 kDa) and ARP2-V5 (54 kDa) in total cell lysates. (**B**) Normalized cell viability% of controls was assessed using MTT assays with 2×10^4^ cells cultured in the absence of serum or transfected with *arp2* cDNA. Viability of these two treatment groups were assayed at 16, 24, 48, and 72 h timepoints. (**C**) Normalized cell viability% of controls assayed using a trypan blue exclusion method. Cells were cultured in the absence of serum or transfected with *arp2* cDNA and assayed at 16, 24, 48, 72, 96, and 120 h timepoints. Mean values are presented (n = 3, X ± S.E.M), ^#^ *p*<0.05, *p<0.01, ^¶^ *p*<0.001 compared to control groups.

Western blot assays show the expression of ARP2-V5 in LNCaP cells first observed 16 h after transfection, with the highest levels of expression detected 72 h post-transfection (Figure 1A). This assay shows bands with molecular weights lower than the one for the fusion protein ARP2-V5 (54 kDa) detected 24, 48, and 72 h post-transfection. These bands are most probably related to degradation products of ARP2-V5 generated during apoptosis.

Normalized cell viability% of controls of LNCaP cells cultured in the absence of serum, as well as LNCaP cells transfected with *arp2* cDNA, was measured using MTT assays. Cell viability was observed to decrease markedly after 16 h, and an additional decrease was seen at 72 h (Figure 1B). A ∼63% decrease in cell viability was observed in *arp2* cDNA-transfected cells at 72 h, which is higher compared to a ∼23% decrease in viability for LNCaP cells cultured in the absence of serum. Therefore, it is very likely that androgen-insensitive LNCaP cells in the absence of serum activate apoptotic mechanisms belatedly with respect to *arp2* cDNA-transfected cells. Normalized cell viability% of controls was also assessed using the trypan blue exclusion method. A mild decrease in viability was detected within the first 24 h for both experimental conditions (Figure 1C). Cell viability further decreased for serum deprived and *arp2* cDNA-transfected cells up to 120 h, with a decrease of ∼44% and ∼42% in cell viability, respectively.

### ARP2 expression and cell viability in CHO cells

**Figure 2:**
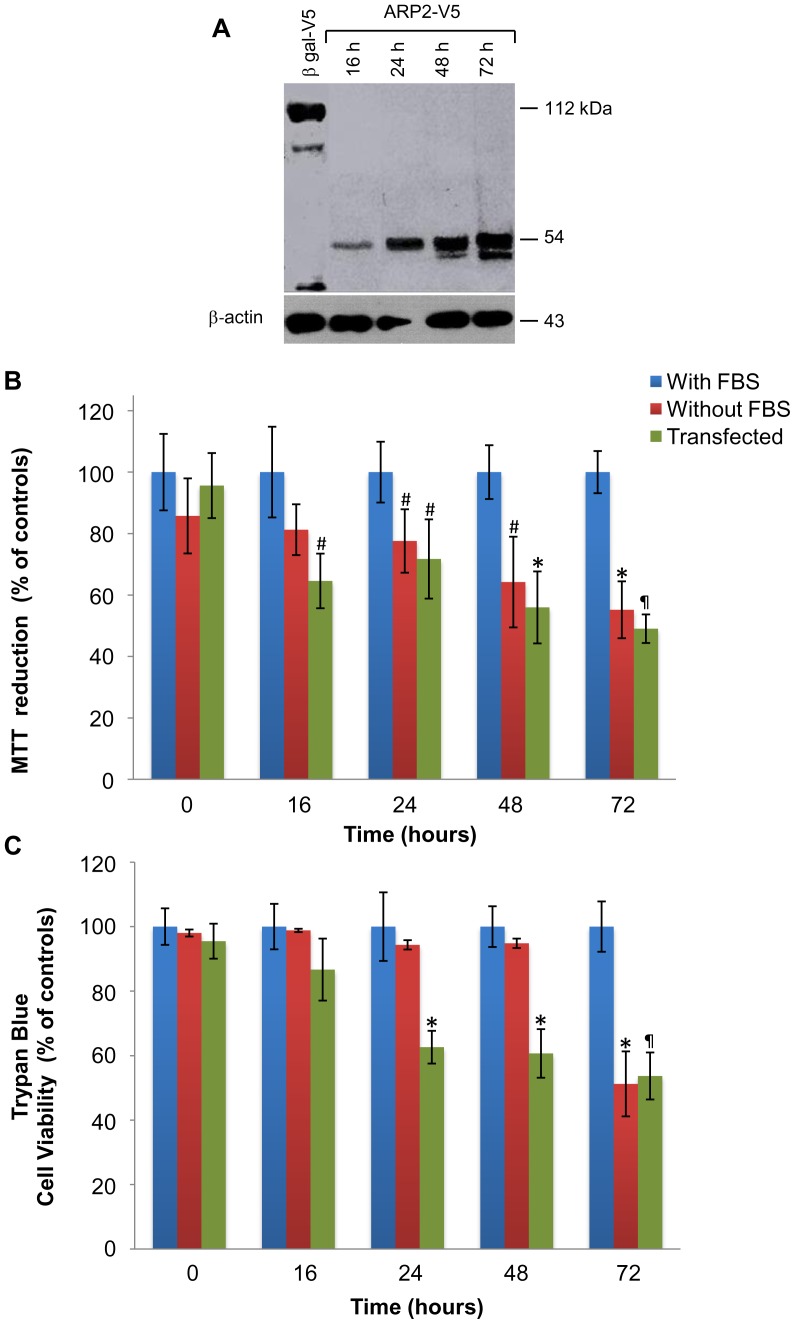
ARP2 expression induces apoptosis in CHO cells. (**A**) Western blots detected expression of β-gal-V5 (112 kDa) and ARP2-V5 (54 kDa) in total cell lysates. (**B**) Normalized cell viability% of controls was assessed using MTT assays with 2×10^4^ cells cultured in the absence of serum or transfected with *arp2* cDNA. Viability of these two treatment groups were assayed at 16, 24, 48, and 72 h timepoints. (**C**) Normalized cell viability% of controls was assessed using a trypan blue exclusion method. Cells were cultured in the absence of serum or transfected with *arp2* cDNA and assayed at 16, 24, 48, and 72 h timepoints. Mean values are presented (n = 3, X ± S.E.M), ^#^ *p*<0.05, *p<0.01, ^¶^ *p*<0.001, compared to control groups.

Western blot assays show the expression of ARP2-V5 in CHO cells first detected at 16 h after transfection, reaching the highest levels 72 h post-transfection. Degradation of ARP2-V5 (54 kDa) shown by a lower molecular weight band detected between 48 and 72 h after transfection, suggests that the apoptotic event is also taking place in this cell type in the same manner as previously shown for LNCaP cells (Figure 2A). It is interesting to point out that the delay in the appearance of these degradation products when CHO cells are compared with LNCaP cells, might be related to the fact that CHO cells constitute an excellent model for the expression of heterologus proteins since protein degradation is kept to a minimum.

During cell viability assays employing reduction of MTT, a constant decrease in viability was observed for CHO cells between 16 h and 72 h of culture. At 72 h, viability decreased by ∼44% for serum deprived cells, while viability of *arp2* cDNA-transfected cells decreased by ∼51% (Figure 2B). Trypan blue cell viability assays show no significant changes in CHO cells observed up to 48 h after serum deprivation. However, a drastic decrease in viability was detected after 72 h incubation, suggesting that up to this time the apoptotic event started to affect the integrity of the plasma membrane. For *arp2* cDNA-transfected cells, a gradual decrease in viability was observed between 16 and 72 h post-transfection. The percentages of viable cells for both groups at 72 h were very similar, with ∼51% viability observed for serum deprived cells and ∼53% viability observed for *arp2* cDNA-transfected cells (Figure 2C). Therefore, similar to LNCaP cells, the cell death index for CHO cells appears to be dependent on the level of ARP2-V5 expression. It is interesting to point out that in the case of *arp2* cDNA-transfected cells in comparison to serum deprived cells, differences obtained between the two assays employed to evaluate cell viability are probable related to the fact that critical metabolic pathways associated to an intracellular calcium overload and the loss of intracellular organelle integrity are primarily affected, before the cell membrane becomes damaged.

### Fura-2 measurement of cytosolic free Ca^2+^ in LNCaP and CHO cells

Since the use of Fura-2 to measure changes in intracellular calcium using whole cell suspensions has been one of the most successful procedures designed for this purpose, LNCaP and CHO cell suspensions were loaded with Fura-2 and [Ca^2+^]i measured. As shown in Table 1, Fura-2 loaded control cells exposed to ionomycin (10 µM) in the presence of a calcium containing buffer, produced in a short period of time an increase in [Ca^2+^]i. Interestingly, LNCaP and CHO *arp2*-transfected cells also showed an increased [Ca^2+^]i demonstrating an enhanced calcium permeability in comparison to control cell lines. Although cells incubated in the absence of fetal bovine serum were also tested, leakage of Fura-2 loaded cells was important and therefore differences between experiments too large to be included in this set of results.

**Table 1:** Intracellular Ca^2+^ concentrations in whole cell suspensions using Fura-2.

Intracellular Ca^2+^ concentrations (nM)						
	CHO			LNCaP		
Time (s)	Control	+ Ionomycin (10 μM)	Transfected Arp2-V5	Control	+ Ionomycin (10 μM)	Transfected Arp2-V5
60	36.0	180.0	169.0	49.0	246.0	250.0
120	40.0	230.0	212.0	56.0	273.0	269.0

Experiments from a series of three different assays. Untreated cells used as controls.

### Apoptosis progression and activation of caspases 3 and 7 in CHO and LNCaP cells

**Figure 3:**
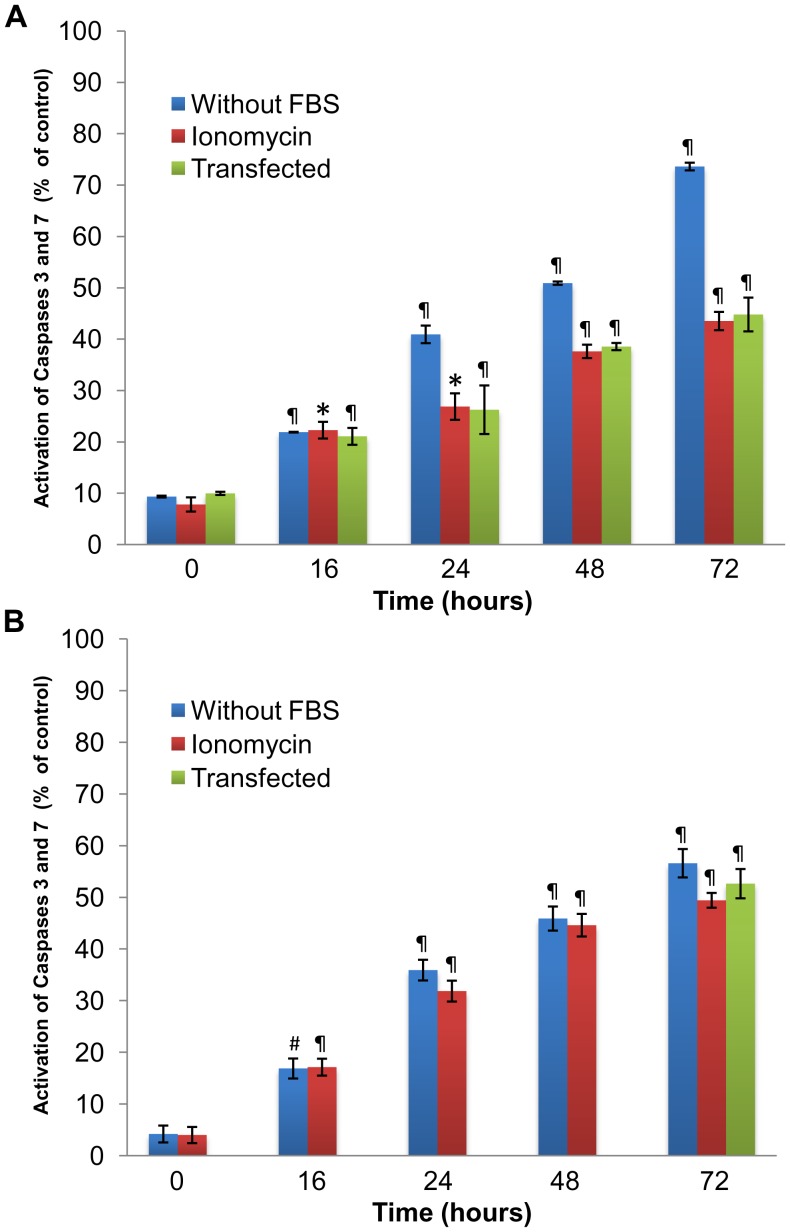
Activity of caspases 3 and 7. Caspase activity was determined using a fluorometric assay employing Z-DEVD-AFC as a substrate. (**A**) CHO cells grown in the absence of serum, transfected with *arp2* cDNA or incubated with 10 µM ionomycin assayed at 16, 24, 48 and 72 h timepoints. (**B**) LNCaP cells grown in the absence of serum, transfected with *arp2* cDNA (72 h) or incubated with 10 µM ionomycin assayed at 16, 24, 48 and 72 h timepoints. Mean values are presented (n = 3, X ± S.E.M), ^#^ *p*<0.05, *p<0.01, ^¶^ *p*<0.001 compared to control groups.

Caspases 3 and 7 classified as effector caspases are activated by caspases 8 and 9 (also known as initiators). Caspases 3 and 7 have also been shown to contribute during apoptosis to the breakdown of substrates such as proteins, nucleic acids and lipids. Figure 3 shows the activity levels detected for caspases 3 and 7. The baseline represents the caspases activity in untreated (control) cells, since the processing of samples took almost 30 min and a certain degree of enzyme activation is always detected. Data are expressed as percentage values relative to the positive control. For serum deprived CHO cells, a gradual activation of caspases 3 and 7 was detected between 24 h and 48 h, with the highest activation levels found after 72 h and activation rates 75% above controls. While *arp2* cDNA-transfected CHO cells or ionomycin (10 µM) treated CHO cells exhibited a gradual increase in caspase activation, the increase observed between 24 and 72 h after transfection or ionomycin treatment was lower than the results obtained with CHO cells incubated in the absence of serum at the same timepoints (Figure 3A). Interestingly, caspases activation in LNCaP cells cultured in the absence of serum, *arp2* cDNA-transfected cells, or ionomycin treated cells, showed the same level of activation, results that correlate well with the above viability data. These results again support the fact that LNCaP cells seem to be more resistant to the onset of an apoptotic event than CHO cells (Figure 3B).

### Subcellular localization

**Figure 4:**
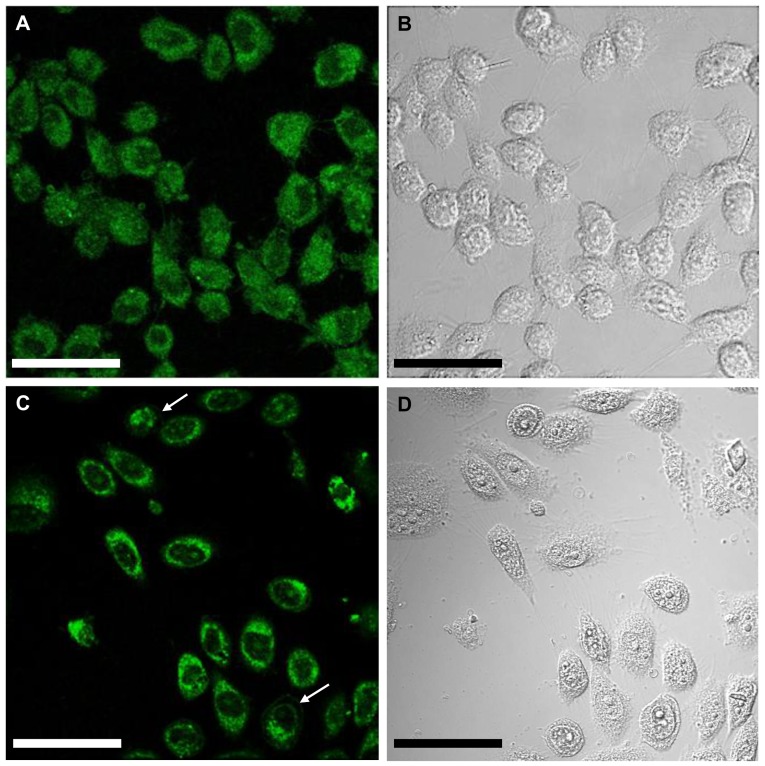
Confocal microscopy of ARP2 fusion protein expressed in CHO cells. CHO cells transfected with e*gfp* cDNA were observed 24 h post-transfection using confocal microscopy (**A**) and DIC microscopy (**B**). CHO cells transfected with *arp2-egfp* cDNA were also observed 24 h post-transfection using confocal microscopy (**C**) and DIC microscopy (**D**). Scale bar 50 µm. White arrows indicate cells that present ARP2 localized to the peri-nuclear region.

**Figure 5:**
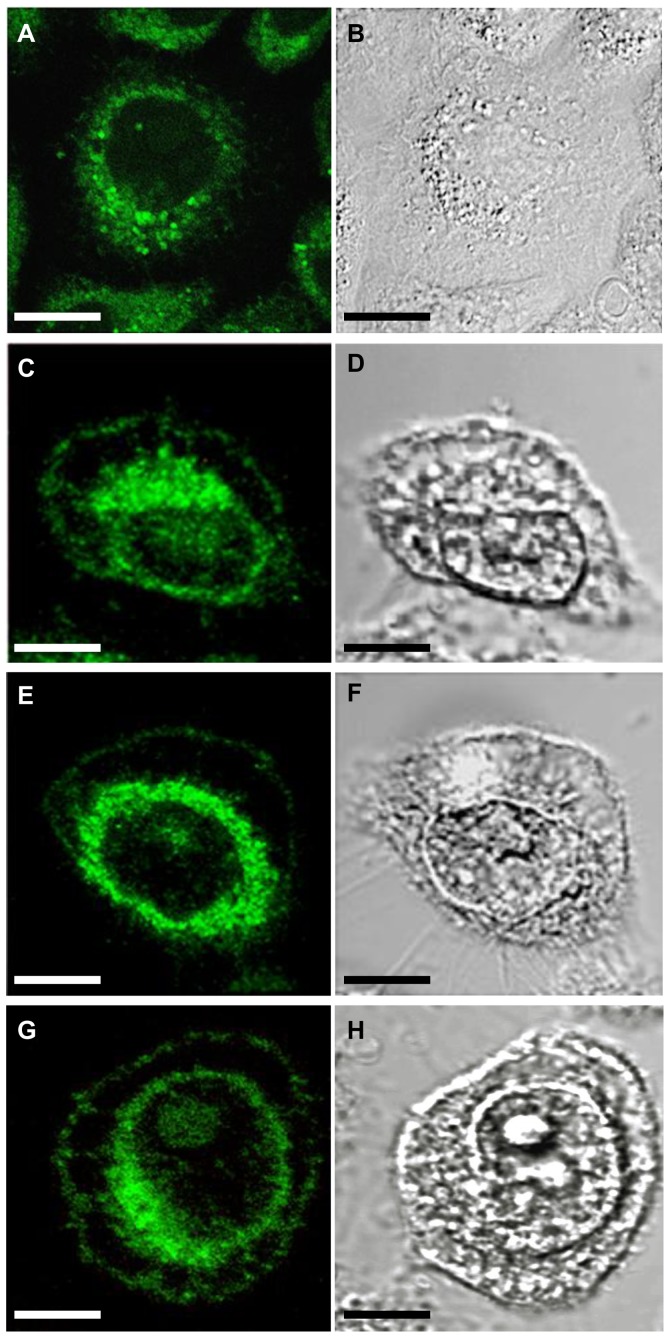
Confocal microscopy of ARP2 fusion protein expressed in CHO cells. CHO cells transfected with *arp2-egfp* cDNA examined at 16, 24, 48 and 72 h post-transfection (**A**, **C**, **E** and **G** respectively). Same using DIC microscopy (**B**, **D**, **F** and **H** respectively). Scale bar 10 µm.

In order to visualize the subcellular localization of ARP2, the expression of an eGFP-labeled-ARP2 fusion protein in the CHO cell line through transfection with *arp2*-*egfp* cDNA and *egfp* cDNA (control) was studied at 24 h post-transfection using a 10× objective lens. Compared to e*gfp* cDNA transfected CHO control cells (Figure 4A), *arp2-egfp* cDNA-transfected cells exhibited a fluorescent signal localized around the nuclear membrane (Figure 4C). The same fields were analyzed using DIC microscopy (Figure 4, B and D). In a single cell analysis of *arp2-egfp* cDNA-transfected CHO cells, a strong fluorescent signal was localized in the peri-nuclear region associated with inner transport vesicles 16 h after transfection (Figure 5A). Between the critical incubation time of 16 and 24 h post-transfection, ARP2-eGFP starts to be localized in the plasma membrane of cells (Figure 5, C, E, and G). DIC microscopy provides important images of the same cells that help to point out that ARP2-eGFP is indeed associated to the region of the plasma membrane (Figure 5, B, D, F, and H). Based on these results and those of our previous studies [15], [40], we hypothesize that following cell damage, an apoptotic event is triggered and ARP2 synthesis activated. From an initial localization concentrated around the nucleus, endoplasmic reticulum vesicles migrate to the plasma membrane and ARP2 becomes a Ca^2+^ permeable channel allowing an important influx of calcium that eventually promotes general membrane damage and cell death.

## Discussion

The results presented in this report, combined with results from our previous studies, support the hypothesis that ARP2 constitutes a Ca^2+^ permeable channel involved in the activation and maintenance of apoptosis. These studies were originally performed in order to identify and characterize Ca^2+^ permeable channels associated with programmed cell death in prostate epithelial androgen-insensitive cancer cells. Employing this cell type, the present study demonstrates the pro-apoptotic role of ARP2 and extends its involvement to other epithelial cell types, such epithelial ovary transformed CHO cells. Although ARP2 has been previously cloned from apoptotic LNCaP cells, this study for the first time shows that transient expression of *arp2* cDNA in this cell type as well as in epithelial ovary transformed CHO cells promotes and maintains apoptosis. Under *in vitro* culture conditions, where phagocytes are normally absent, apoptotic cells are lysed in a process similar to necrosis, termed “secondary necrosis” or “post-apoptotic necrosis” [19]. It is at this point that dead cells are detected by dye diffusion to the cytoplasm. In this respect it is interesting to mention that during the course of this investigation, two methods employed to measure cell viability, MTT reduction and trypan blue exclusion, might not be a reflection of each other. While *arp2* cDNA-transfected CHO cells followed a fairly similar pattern of viability when measured by these two methods, *arp2* cDNA-transfected LNCaP cells clearly show differences when viability is measured by MTT reduction or by trypan blue exclusion. An explanation might be found if we visualize apoptotic CHO cells having cytoplasmic organelles damaged first by a calcium overload that would for instance affect mitochondrial respiration, and only when calcium reaches a critical [Ca^2+^]i threshold, the plasma membrane is damaged leading to a post-apoptosis necrotic condition that would allow trypan blue inclusion into the cytoplasm.

Biochemical evidence of apoptosis was further demonstrated with the detection of an increase in activity of caspases 3 and 7 in both cell types studied, independently if cells were grown in the absence of serum, transfected with *arp2* cDNA or incubated in presence of ionomycin. Although caspases activation was found to increase at the same rate in the two cell lines when *arp2* cDNA transfection and ionomycin were tested, incubation in the absence of serum showed a substantial difference. The fact that under this condition caspases activation in the CHO cell line reached levels of 75% in comparison of an average of 50% activation in the two cell lines with the other two methods, supports the fact that CHO cells seem to reach a critical [Ca^2+^]i threshold as discussed above. Experiments performed with ionomycin support the notion that an influx of calcium that would increase [Ca^2+^]i is able to activate caspases activity and support an apoptotic event. It has recently been demonstrated that transfection of LNCaP cells with Orai1, a calcium release activated calcium (CRAC) channel, reestablishes the store-operated Ca^2+^ entry, as well as the ability of these cells to trigger apoptosis in response to different inducers [48]. As such, transfection of LNCaP cells with *arp2* cDNA could reestablish Ca^2+^ entry currents through the plasma membrane and also trigger apoptosis. In androgen-sensitive cells, Ca^2+^ release from internal stores in the endoplasmic reticulum has been also shown to be an activator of apoptosis [35]. Nevertheless, it has been reported that the release of Ca^2+^ from internal stores in androgen-insensitive cells, *per se*, might not be sufficient to induce programmed cell death [49]–[51]. Consequently, the identification and characterization of ARP2 as a Ca^2+^ permeable channel located in the plasma membrane could explain the sustained Ca^2+^ influx necessary to maintain a high apoptosis-activation level in epithelial cancer cells. This scenario is supported by the fact that Fura-2 loaded *arp2* cDNA-transfected cells, when placed in a calcium containing buffer, start to show in a short period of time increased intracellular calcium concentrations in comparison to ionomycin treated control cells. Depending on the cytoplasmic calcium buffering capacity of cells, this type of event might have different ways to be modulated. In this regard, LNCaP cells have been reported to present an interesting resistance to the deleterious effects of sudden [Ca^2+^]i increments [49], [51]. Therefore, androgen-insensitive LNCaP cells under physiological conditions might be able to block the signaling cascades and/or mechanisms that induce ARP2 expression. However, induction of apoptosis in these cells using non-physiological methods, such as exposure to ionomycin or serum deprivation may activate alternative mechanisms for the expression and proper functioning of ARP2 [40]. For example, transfection of LNCaP cells and CHO cells with *arp2* cDNA generally resulted in lower levels of viability compared to serum deprived cells, thereby suggesting that ARP2 overexpression can accelerate apoptosis.

Our confocal microscopy results in combination with the analysis of secondary structure and hydrophobicity profiling which shows four transmembrane domains support the idea that ARP2 corresponds to a transmembrane protein [40].

Based on the results, we propose that ARP2 represents a cationic channel overexpressed during the influx of extracellular Ca^2+^, necessary for the activation of apoptosis. Evidence for this hypothesis includes: 1) Identification and electrophysiologically characterization of this novel Ca^2+^-permeable channel activated during apoptosis in LNCaP cells [15]. 2) Expression of TRPC1 like channels in apoptotic LNCaP cells in relation to cells that do not undergo apoptosis [40], [52], [53]; 3) A high homology between htrp3 channels and the N- and C- terminal regions of ARP2; 4) A substantial increase of *arp2* mRNA levels in apoptotic LNCaP cells compared to non-apoptotic cells; 5) Induction of inward Ca^2+^ currents through the cell membrane and premature death when ARP2 is expressed in *Xenopus laevis* oocytes [40]; 6) Induction of apoptosis in mammalian cells with ARP2 expression; 7) *In silico* defined transmembrane properties of ARP2.

On the other hand, we have interestingly found that ARP2 shares a high homology with a region of Prp8, a protein that plays an important role in the assembly of spliceosome [54]–[56]. Accordingly, it might be hypothesized that during the executor stage of apoptosis (e.g., the third stage), ARP2 (50 kDa) might be released from Prp8 (274 kDa) by protease digestion, and therefore be able to acquire an alternative function as a Ca^2+^ permeable channel involved in the activation of apoptosis. A second possibility might be consistent with the fact that *arp2* mRNA could be generated following alternative splicing of the Prp8 gene (e.g., during the second stage of apoptosis) [57]. A third possibility might be that *arp2* mRNA is synthesized from its own gene through promoter activation. Although the relationship between the pro-apoptotic activity of ARP2 and the potential role of Prp8 involved in the processing of mRNA might be feasible, further studies are necessary to dilucidate this phenomenon.

In conclusion, over-expression of ARP2 in epithelial transformed cells such as epithelial prostate cancer cells and epithelial ovary transformed cells, constitutes a novel mechanism to accelerate apoptosis by allowing a sudden increase in [Ca^2+^]i when ARP2 becomes incorporated in the cell membrane acting as a channel-like protein.
